# First Record of *Aspergillus fijiensis* as an Entomopathogenic Fungus against Asian Citrus Psyllid, *Diaphorina citri* Kuwayama (Hemiptera: Liviidae)

**DOI:** 10.3390/jof8111222

**Published:** 2022-11-19

**Authors:** Jianquan Yan, Hao Liu, Atif Idrees, Fenghao Chen, Huilin Lu, Gecheng Ouyang, Xiang Meng

**Affiliations:** 1 Guangdong Key Laboratory of Animal Conservation and Resource Utilization, Guangdong Public Laboratory of Wild Animal Conservation and Utilization, Institute of Zoology, Guangdong Academy of Science, Guangzhou 510260, China; 2 College of Plant Protection, South China Agricultural University, Guangzhou 510642, China

**Keywords:** *Diaphorina citri*, entomopathogenic fungi, *Aspergillus fijiensis*, biological control, bioassay

## Abstract

The Asian citrus psyllid *Diaphorina citri* Kuwayama (Hemiptera: Liviidae) is the most widespread and devastating pest species in citrus orchards and is the natural vector of the phloem-limited bacterium that causes Huanglongbing (HLB) disease. Thus, reducing the population of *D. citri* is an important means to prevent the spread of HLB disease. Due to the long-term use of chemical control, biological control has become the most promising strategy. In this study, a novel highly pathogenic fungal strain was isolated from naturally infected cadavers of adult *D. citri*. The species was identified as *Aspergillus fijiensis* using morphological identification and phylogenetic analysis and assigned the strain name GDIZM-1. Tests to detect aflatoxin B_1_ demonstrated that *A. fijiensis* GDIZM-1 is a non-aflatoxin B_1_ producer. The pathogenicity of the strain against *D. citri* was determined under laboratory and greenhouse conditions. The results of the laboratory study indicated that nymphs from the 1st to 5th instar and adults of *D. citri* were infected by *A. fijiensis* GDIZM-1. The mortality of nymphs and adults of *D. citri* caused by infection with *A. fijiensis* increased with the concentration of the conidial suspension and exposure time, and the median lethal concentration (LC_50_) and median lethal time (LT_50_) values gradually decreased. The mortality of *D. citri* for all instars was higher than 70%, with high pathogenicity at the 7th day post treatment with 1 × 10^8^ conidia/mL. The results of the greenhouse pathogenicity tests showed that the survival of *D. citri* adults was 3.33% on the 14th day post-treatment with 1 × 10^8^ conidia/mL, which was significantly lower than that after treatment with the *Metarhizium anisopliae* GDIZMMa-3 strain and sterile water. The results of the present study revealed that the isolate of *A. fijiensis* GDIZM-1 was effective against *D. citri* and it provides a basis for the development of a new microbial pesticide against *D. citri* after validation of these results in the field.

## 1. Introduction

The Asian citrus psyllid *Diaphorina citri* Kuwayama (Hemiptera: Liviidae) is a major pest of *Citrus reticulata* Blanco, *Murraya exotica* Jack and other Rutaceae plants [1]. It is also the main transmission vector of citrus Huanglongbing (HLB), which causes huge economic losses to citrus production [2,3]. *D. citri* has a long lifespan, rapid reproduction and severe generation overlap [4]. At present, chemical pesticides are still considered to be the main means of controlling *D. citri* [5]. However, the long-term and large-scale use of chemical pesticides will not only increase the resistance of *D. citri* but also cause environmental pollution, imbalance of the field community structure and other negative effects on the ecosystem [6,7,8]. Therefore, it has become a new research priority to find an environmentally friendly approach for the management of *D. citri* and to improve the sustainable development of the citrus industry.

Entomopathogenic fungi are pathogenic micro-organisms that can infect and penetrate the host and cause disease, which ultimately leads to the death of insects [9]. In the theory of integrated pest management (IPM), entomopathogenic fungi have become one of the most critical tools in the population control of agricultural pests [10,11,12]. Under natural conditions, entomopathogenic fungi mainly adhere to the surface of the host through spores. Under an appropriate temperature and humidity, conidia absorb water and germinate and differentiate into appressoria and penetration pegs to provide mechanical pressure. At the same time, they also secrete chitinase, protease, lipase and other hydrolases to help hyphae to penetrate the host cuticle and endodermis into the hemolymph [13,14,15]. The mycelial proliferates in the hemocoel, consuming the nutrients in the host, weakening the host insects, reducing their resistance, and producing metabolic toxins, such as destruxin and ciclosporin, inhibiting the cell-mediated immunity defense system of the host, reducing the activity of detoxification enzymes in humoral immunity, destroying the cell morphological structure and physiological function of the host, causing death [16,17,18]. After the death of the insect, entomopathogenic fungi will continue to grow until the hyphae invade all of the tissues and organs and penetrate the host cuticle to produce conidia [12]. Then, the conidia form a new infection cycle by natural transmission, infecting other hosts [19,20].

Entomopathogenic fungi are the largest group of entomopathogenic micro-organisms [21]. They have the advantages of widely existing, a broad-spectrum action, long duration of efficacy, relatively safe for nontarget organisms and less likelihood of the pest developing resistance [22]. Entomopathogenic fungi have become an important resource for controlling agricultural and forestry pests due to their unique insecticidal methods and high efficiency of epidemic potential [9,23]. Currently, more than 1000 species have been reported. For example, *Metarhizium anisopliae*, *Beauveria bassiana* and *Cordyceps fumosorosea* have good control effects on *Locusta migratoria* Meyen, *Aphis gossypii* Glover, *Spodoptera frugiperda* and *Trialeurodes vaporariorum* Westwood and have been applied to the green control of agricultural pests [9,24,25,26]. In recent years, a large number of entomopathogenic fungi have been reported for the control of *D. citri*. *Paecilomyces variotii*, *Hirsutella citriformis* and *Akanthomyces lecanii* have the ability to infect *D. citri* [27,28,29,30]. In addition, *B. bassiana* and *M. anisopliae*, which have been commercially produced and applied, can also be used for the control of *D. citri*. The existing experimental results showed that these two kinds of entomopathogenic fungi could significantly reduce the population density of *D. citri* and achieve the green control effect of using entomopathogenic fungi to control insects [31,32]. Although some of the entomopathogenic fungi described above have been reported to be effective against *D. citri*, there is also a need to find new sources of entomopathogenic fungi to develop biological control methods for *D. citri*. However, there are few reports on the pathogenicity of *Aspergillus* species against *D. citri*. Therefore, screening strains with high pathogenicity against *D. citri* is of great significance for the field control of *D. citri* and the development of fungal insecticides in the future.

*Aspergillus* species are diverse fungi that are widely distributed in nature. The most recent research indicates that *Aspergillus* species are available for the biological control of *L. migratoria*, *Spodoptera litura* and *Dolichoderus thoracicus*, including *Aspergillus flavus*, *Aspergillus nomius* and *Aspergillus oryzae* [33,34,35]. In this study, we collected a fresh, naturally infected adult *D. citri* cadaver in a lemon orchard. The main aims of our work were (a) to isolate and identify a novel entomopathogenic fungus from *D. citri*, (b) to detect its production of aflatoxin B_1_, (c) to study its biological characteristics and determine its pathogenicity against *D. citri*, and (d) to provide a biological control strategy for *D. citri*. This study is expected to provide a useful reference for the biological control of *D. citri*., and to provide a reasonable theoretical basis and technical support for the comprehensive management of pests.

## 2. Materials and Methods

### 2.1. D. citri Cadaver Collection and Isolation of Aspergillus Species

A fresh, naturally infected cadaver *D. citri* adult was collected from a leaf in a lemon orchard, which was located on Maofeng Mountain (113°46′49″ E, 23°29′11″ N), Baiyun District, Guangzhou, China. The *D. citri* cadaver was infiltrated in 70% ethanol for 30 s, washed with sterile water three times and dried with sterile filter paper. Then, the *D. citri* cadaver was placed on PDA medium in a biochemical incubator for 1–2 days at 25 ± 1 °C in the dark until mycelia grew around the insect body and then the mycelia were selected and transferred to a new PDA plate for culture. Then, a monoconidial culture was obtained. This isolated and purified strain was designated “GDIZM-1” and deposited in Guangdong Microbial Culture Collection Center (GDMCC) with the conservation number GDMCC 62135.

The GDIZM-1 strain was plated on potato dextrose agar (PDA) medium and cultured in an incubator for 10 days at 25 ± 1 °C. The fungal conidia were suspended in 10 mL sterile water containing 0.1% Tween 80 (*v*/*v*), and the conidia were evenly dispersed by magnetic stirrers for 30 min to break up the conidial clumps to ensure a homogenous suspension. The conidial suspension was then filtered. After treatment, an optical microscope (ZEISS, Axio Imager 2, Germany) was used to calculate the total conidial germination rate of the conidia, which should be above 95% [36]. Then, the conidial suspension was adjusted to 1 × 10^7^ conidia/mL suspension.

The adult and nymph of *D. citri* were immersed in a 1 × 10^7^ conidia/mL suspension for 2 s. The excess conidial suspension was dried with sterile filter paper and the insects were transferred to young leaves. The *D. citri* were separately reared in an incubator (25 ± 1 °C, 75 ± 5% RH, L:D = 14:10). The infection and spore growth on the 1st- to 5th-instar nymphs and adults of *D. citri* were observed and recorded with a stereomicroscope (ZEISS, SteREO Discovery. V20, Germany) and identification was based on the phenotypic characteristics and morphology of the mycelia and conidia grown from *D. citri* [37,38].

### 2.2. Morphological Observation

To measure the growth rate and conidial yield, the GDIZM-1 strain was first cultivated on SDAY medium at 27 °C for 10 days. The colony diameter was measured daily. Then, the conidia were collected by filtration from a water suspension containing 0.1% Tween 80 (v/v), and quantified using a hemocytometer. Lactophenol cotton blue staining was used to prepare the slides, and the conidial and sporulation structures of the strain were observed at 100× magnification under an optical microscope (ZEISS, Axio Imager 2, Germany). Both examinations were replicated three times.

### 2.3. DNA Extraction and Phylogenetic Analysis of the GDIZM-1 Strain

Total DNA of the GDIZM-1 strain was isolated from samples of the test strains using a fungal DNA kit, following the manufacturer’s instructions (Fungal DNA Kit; Sangon Biotech, Shanghai, China). The DNA-specific sequence of this study consists of three genes: internal transcribed spacer (ITS), translation elongation factor 1-α (TEF1-α) and RNA polymerase II second largest subunit (RPB2). The purified DNA specimens were amplified with primers ITS4-F (5′-TCCTCCGCTTATTGATATGC-3′), ITS5-R (5′-GGAAGTAAAAGTCGTAACAAGG-3′); EF-1983-F (5′-GCYCCYGGHCAYGGTGAYTYAT-3′), EF-12218-R (5′-ATGCACCRACRGCRACRGTYTG-3′); fRPB2–5F (5′-GAYGAYMGWGATCAYTTYGG-3′), fRPB2–7cR (5′-CCCATRGCTTGYTTRCCCAT-3′) [39,40,41,42]. Each PCR mixture (50 μL) contained 25 µL 2 × Ultra Taq PCR MasterMix, 1 µL each primer, 1 µL DNA, and 22 µL ddH2O (TaKaRa, Kusatsu, Shiga, Japan). ITS gene sequence amplification was performed with an initial denaturation of 3 min at 94 °C followed by 35 cycles of 30 s at 94 °C, 30 s at 55 °C, and 45 s at 72 °C and a final extension of 10 min at 72 °C. The TEF1-α gene sequence amplification was performed with an initial denaturation of 10 min at 95 °C followed by 40 cycles of 30 s at 94 °C, 30 s at 55 °C, and 1 min at 72 °C and a final extension of 10 min at 72 °C. The RPB2 gene sequence amplification was performed with an initial denaturation of 10 min at 95 °C followed by 40 cycles of 30 s at 94 °C, 30 s at 50 °C, and 1 min at 72 °C and a final extension of 10 min at 72 °C. The PCR products were separated by 1.0% agarose gel electrophoresis, stained with Gold View in 1 × TAE buffer (Sangon, Shanghai, China), and photographed under UV light. Then, the target PCR products were sent to The Beijing Genomics Institute (BGI; Shenzhen, China) for complete sequencing with PCR primers.

The resulting sequences were checked and aligned using Lasergene v7.1 (DNASTAR, Inc., Madison, Wisconsin USA). The ITS, TEF1-α and RPB2 similarity of the sequences were compared with other fungal homologous sequences (Table 1) using the “BLAST” tool on the National Center for Biotechnology Information website (https://blast.ncbi.nlm.nih.gov/Blast.cgi, accessed on 15 March 2022). Based on the ITS, TEF1 and RPB2 marker genes, the phylogenetic tree was constructed using the maximum likelihood (ML) method of MEGA7: Molecular Evolutionary Genetics Analysis version 7.0 for bigger datasets (Kumar et al. 1994) [43], Institute of Molecular Evolutionary Genetics, Pennsylvania State University, USA. Node support was assessed using a bootstrap procedure of 1000 replicates [44,45]. *Emericella acristata* strains were used as the outgroup in the phylogenetic analysis.

### 2.4. Detection of Aflatoxin B_1_

The presence of aflatoxin B_1_ was detected by a competitive enzyme-linked immunosorbent assay, according to the manufacturer’s instructions provided with the aflatoxin B_1_ test kit (EKT-010, Pribolab, Qingdao, China). Extraction and detection were performed according to the manufacturer’s instructions. The GDIZM-1 strain was cultured on PDA medium in an incubator for 10 days at 25 ± 1 °C followed by preparation of 1 × 10^8^ conidia/mL suspension. Sterilized Czapek’s broth, 100 mL, (containing/L: K_2_HPO_4_ 1 g, FeSO_4_ 0.01 g, Na_2_SO_4_ 3 g, sucrose 30 g, MgSO_4_ ∙7H_2_O 0.5 g, and KCl 0.5 g), added to Erlenmeyer flasks (250 mL), was inoculated with 1 mL of conidial suspension of GDIZM-1 strain followed by incubation at 150 rpm and 27 °C for 1 week following Wu et al. [46]. A culture of the aflatoxin B_1_ producer *A. flavus* ATCC 28,539 grown in Czapek’s broth was used as a control. After 1 week of growth, fermentation broth (1 mL) was extracted with 10 mL of methanol for 10 min. Then, mixture was centrifuged in an Eppendorf 5804R centrifuge (Eppendorf, Framingham, MA, USA) at 10,000 rpm for 10 min, and the resultant supernatant was collected. Afterward, 200 μL of clear extract diluted with 800 μL of sample dilution buffer was directly subjected to detection of aflatoxin B_1_ [47,48]. The optical densities (OD) were measured at 450 nm using an MPP spectrophotometer (PowerWave HT, BioTek, Winooski, USA). The content of aflatoxin B_1_ in the fermentation broth can be determined by comparing the OD value of the sample with the OD value of the standard product provided by the kit. All standard solutions and sample solutions were analyzed in triplicate wells on a plate, and the whole experiment was conducted three times.

### 2.5. Plants and Insects for Testing

The *Murraya paniculata* (L) jack plants used in this study were purchased from Chentian Nursery, Baiyun district, Guangzhou, China and potted in a greenhouse (temperature 25 ± 1 °C, 75 ± 5% RH, L:D =14:10) with nutrient soil as the substrate (perlite:vermiculite:nutrient soil = 1:1:3). These plants were regularly fertilized, watered and pruned.

The colony of *D. citri* was collected from the campus of Sun Yat-sen University and transferred to *M. paniculata* seedlings in a greenhouse at the Institute of Zoology, Guangdong Academy of Sciences. Nymphs of *D. citri* were divided into younger nymphs (1st-2nd instar), middle nymphs (3rd-4th instar) and older nymphs (5th instar) according to their morphological characteristics under a stereomicroscope for uniformity in instar before use in bioassays.

### 2.6. Pathogenicity Test

#### 2.6.1. Laboratory Bioassays on *D. citri*

Experiments were performed in an incubator (25 ± 1 °C, 75 ± 5% RH, L:D = 14:10). Six life stages of *D. citri* were used: younger nymphs (1st-2nd instar), middle nymphs (3rd-4th instar) and older nymphs (5th instar) and mature adults, which were selected for the bioassays. The conidial suspensions of the GDIZM-1 strain were diluted to five concentrations with sterile distilled water (1 × 10^4^, 1 × 10^5^, 1 × 10^6^, 1 × 10^7^, and 1 × 10^8^ conidia/mL). Sterile water containing 0.1% Tween 80 was used as a control (ck). The 20 insects of the different developmental stages of *D. citri* were immersed in each concentration of conidial suspension or sterile water for 2 s. The excess conidial suspension was dried with sterile filter paper and the insects were transferred to young leaves of *M. paniculata* and bagged. Each test was replicated three times. The *D. citri* infected by the GDIZM-1 strain were observed daily. The dead individuals were removed and placed in sterilized Petri dishes to promote fungal growth, and whether it was caused by fungal infection by the GDIZM-1 strain was determined.

#### 2.6.2. Greenhouse Bioassays on *D. citri*

The pathogenicity determination of the GDIZM-1 strain against *D. citri* adults was performed in a greenhouse. The *Metarhizium anisopliae* GDIZMMa-3 strain, which we have already preserved in the laboratory of the Institute of Zoology, Guangdong Academy of Science, was used as a positive control. The conidial suspensions of the GDIZM-1 strain and *M. anisopliae* GDIZMMa-3 strain were diluted with sterile water to 1 × 10^8^ conidia/mL. A total of 20 *D. citri* adults were evenly sprayed with the individual conidial suspensions or sterile water containing 0.1% Tween 80 as a control (ck) in each bioassay, with each assay replicated three times. The *D. citri* infected by the two entomopathogenic fungi were observed daily, and the dead individuals were removed and placed in sterilized Petri dishes filled with moist filter paper to promote fungal growth and confirm the mortality was due to infection by the fungal isolates.

### 2.7. Data Analysis

The mortality and survival of *D. citri* after exposure to the tested strains were calculated. The GDIZM-1 strain was used to determine their LC_50_ and LT_50_ values on the nymphal stage and adults of *D. citri* using a biological assay procedure of probit regression analysis with statistical software SPSS 25.0 [49]. They were subjected to one-way analysis of variance (ANOVA) using Duncan’s highly significant difference test at a 95% level of significance. SPSS 25.0 software was used to perform the data analysis and to calculate the homogeneous letters. The results were considered to be statistically significant when *p* values were <0.05. Student’s t test was used to analyze the differences in the OD values in aflatoxin B_1_ detection experiments and the mortality difference of *D. citri* at different developmental stages after treatment with various concentrations of the *A. fijiensis* GDIZM-1 strain.

## 3. Results

### 3.1. Morphological Identification of Infected D. citri

**Figure 1:**
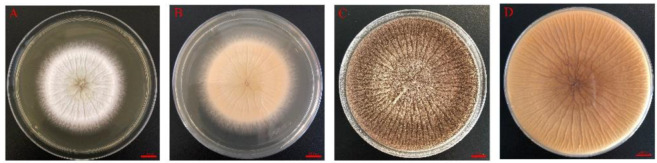
The morphology of the *A. fijiensis* GDIZM-1 strain on SDAY. (**A**,**B**) Colony of the *A. fijiensis* GDIZM-1 strain on SDAY for 3 days; (**C**,**D**) Colony of the *A. fijiensis* GDIZM-1 strain on SDAY for 10 days. Scale bar of A, B, C and D = 10 mm.

**Figure 2:**
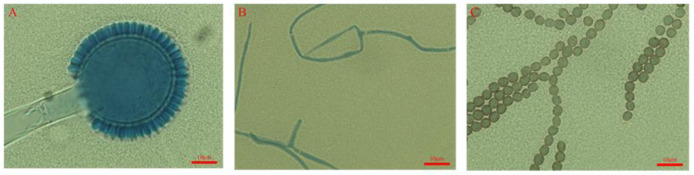
The microscopic morphology of the *A. fijiensis* GDIZM-1 strain on SDAY. (**A**) Conidiophores and uniseriate globose vesicles; (**B**) Podocytes; (**C**) Conidial chains. Scale bar of (**A**–**C**) = 10 μm.

The morphological characteristics of the GDIZM-1 strain are shown in Figure 1. The fungal colonies had radial grooves. The mycelial texture was dense and flat. The frontal side of the colony was white in the early stage and tawny and powdery in the late stage. The color of the mature colony gradually deepened and became earthy yellow (Figure 1). The diameter of the *A. fijiensis* strain colony was 83.70 mm after 5 days of incubation on SDAY, and the sporulation was 3.86 × 10^8^ conidia/mL after 10 days of incubation (Table 2). The conidiophores had podocytes. The conidiophores were straight with a size of 200–1100 μm × 8–16 μm and inflated to uniseriate globose vesicles with a diameter of 20–60 μm on the top. The conidia were coarsely ellipsoidal to slightly fusiform with a diameter of 3–5 μm and linked into a chain (Figure 2).

**Figure 3:**
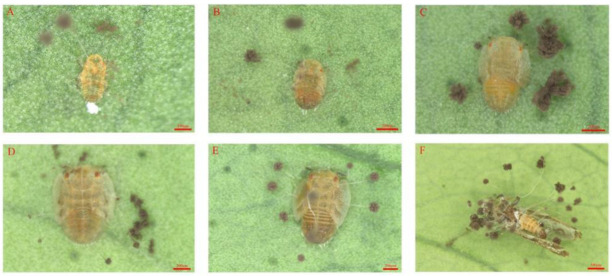
The infection phenotype of *D. citri* nymphs and adults with the *A. fijiensis* GDIZM-1 strain (1 × 10^7^ conidia/mL). Panels (**A**–**F**) are the 1st, 2nd, 3rd, 4th and 5th nymphs and mature adults of *D. citri* on the 10th day after infection. Scale bar of (**A)** = 100 μm; scale bars of (**B**–**E**) = 200 μm; scale bar of (**F**) = 500 μm.

The infection test using *D. citri* nymphs and adults in the laboratory showed that the insects moved slowly and suffered a slight spasm during the early stages of infection. Infected adult *D. citri* clung tightly to leaves until they were completely covered with hyphae and finally died. Microscopic observation showed hyphae and conidia growing from the intersegmental membranes of the leg and abdomen of infected *D. citri* after 48–72 h. Then, *D. citri* was wrapped by mycelia, including the antennae and wings, after 10 days of infection. The morphological identification and infection observation indicated that the GDIZM-1 strain was *A. fijiensis* (Figure 3).

### 3.2. Sequencing and Phylogenetic Analysis

**Figure 4:**
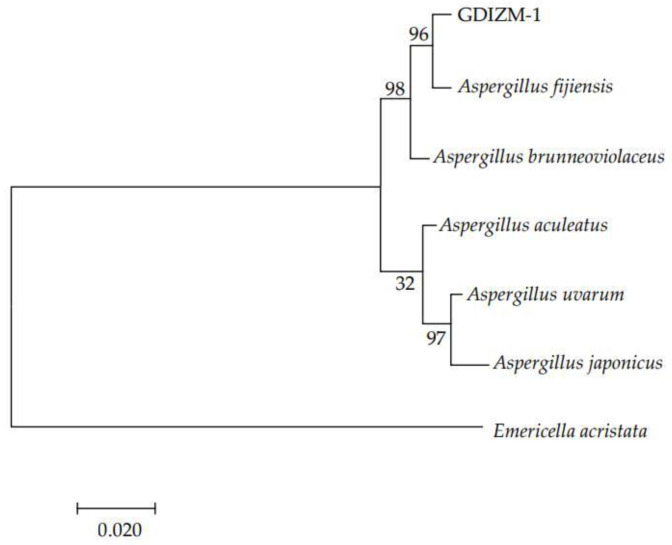
Maximum likelihood (ML) phylogram of the concatenated alignment of ITS, TEF1, and RPB2 sequences for the GDIZM-1 strain. *E. acristata* was used as the outgroup. The percentage of replicate trees in which the associated taxa clustered together in the bootstrap test (1000 replicates) are shown next to the branches.

DNA fragment sequencing results showed that the ITS gene, TEF1 gene and RPB2 gene of the GDIZM-1 strain were 549 bp, 802 bp and 942 bp, respectively. The DNA sequences were then submitted to GenBank, where they were assigned the accession numbers OM925539, ON000912 and ON000911. The DNA sequences by BLAST comparison in GenBank showed that the GDIZM-1 strain was 99~100% homologous to the *A. fijiensis* strain. The ITS gene, TEF1 gene and RPB2 gene sequences were concatenated to construct a neighbor-joining tree. Phylogenetic analysis indicated that the GDIZM-1 strain clustered with the *A. fijiensis* strain clade (Figure 4), which supported our morphological identification that the GDIZM-1 isolate is an *A. fijiensis* strain.

### 3.3. Aflatoxin B_1_ Detection

**Figure 5:**
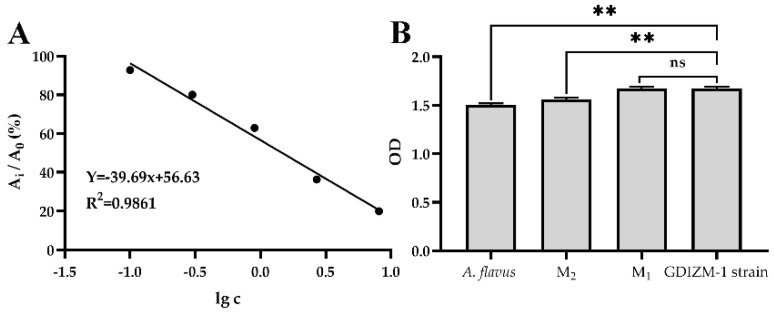
Standard curve and aflatoxin B_1_ in the metabolites of the GDIZM-1 strain. (**A**). A_i_ represents the OD value of aflatoxin B_1_ contained in each aflatoxin standard (0.1, 0.3, 0.9, 2.7, 8.1 ppb); A_0_ represents the OD value of aflatoxin B_1_ in the standard containing 0 ppb of aflatoxin B_1_; lgc represents the logarithm of the concentration. (**B)**. Comparisons of the optical density (OD) values of aflatoxin B_1_ by aflatoxin B_1_-producing *A. flavus* ATCC28539 grown in CA broth for 1 week (*A. flavus*) and the GDIZM-1 strain grown under the same conditions. Standard solutions M_1_ and M_2_ contained 0 ppb and 0.1 ppb aflatoxin B_1_. The OD values were obtained using ELISA, according to the aflatoxin B_1_ test kit manufacturer’s instructions. Data represent the means ± SEs from three replicates, each of which used three wells on the plate. Asterisks and NS indicate significant and nonsignificant differences, respectively, as determined by t tests (** = *p* < 0.01; ns = *p* > 0.05).

The standard curve showed a good negative linear relationship between the optical density (OD) values and the concentration of aflatoxin B1 such that the presence of aflatoxin B1 in the sample lowered the OD values, indicating that the detection method used is feasible (Figure 5A). There was no significant difference between the OD values of the metabolite produced by the GDIZM-1 strain grown in Czapek’s broth for 1 week and that of aflatoxin B_1_ in the standard solution containing 0 ppb aflatoxin B_1_. In contrast, the OD values of the aflatoxin B1 producer *A. flavus* ATCC28539 and that of aflatoxin B_1_ in the standard solution containing 0.1 ppb of aflatoxin B_1_ were extremely significantly lower when grown under the same conditions as the GDIZM-1 strain (Figure 5B). Based on the above results, we believe that *A. fijiensis* GDIZM-1 is a non-aflatoxin B_1_ producer.

### 3.4. Pathogenicity Analysis of the GDIZM-1 Strain against D. citri

#### 3.4.1. Pathogenicity Determination in the Laboratory

**Figure 6:**
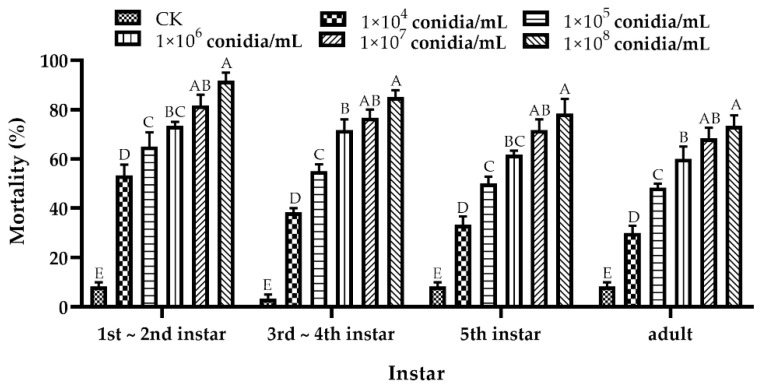
The mortality (means ± SEs) of *D. citri* at different developmental stages treated with various concentrations of the *A. fijiensis* GDIZM-1 strain. The data in the figure are the mortality of *D. citri* on the 7th day after infection. One-way ANOVA and Duncan’s new multiple range method were used to analyze the differences among the different treatments. Different capital letters indicate that the difference was extremely significant (*p* < 0.01).

The bioassay results showed that the *A. fijiensis* GDIZM-1 strain had high pathogenicity to both nymphs and adults of *D. citri*. The mortality of nymphs and adults of *D. citri* gradually increased with an increasing conidial concentration (Figure 6). The LC_50_ values of the nymphs and adults of *D. citri* were different, from high to low: adult > older nymphs (5th instar) > middle nymphs (3rd-4th instar) > younger nymphs (1st-2nd instar) (Table 3). When treated with a low concentration (1 × 10^5^ conidia/mL) of the *A. fijiensis* GDIZM-1 strain, after 7 days the mortality of the nymphs and adults of *D. citri* was more than 45%, which was significantly higher than that of the control (younger nymphs, t = 9.430, *p* = 0.001; middle nymphs, t = 15.500, *p* < 0.001; older nymphs, t = 12.500, *p* < 0.001; adult nymphs, t = 16.971, *p* < 0.001). With the increase in conidial concentration, the mortality of *D. citri* increased, and the mortality of the nymphs and adults of *D. citri* was more than 70% after 7 days when treated with the *A. fijiensis* GDIZM-1 strain with 1 × 10^8^ conidia/mL, which was significantly higher than that of the control (younger nymphs, t = 22.361, *p* < 0.001; middle nymphs, t = 24.500, *p* < 0.001; older nymphs, t = 11.225, *p* < 0.001; adult nymphs, t = 13.789, *p* < 0.001).

**Figure 7:**
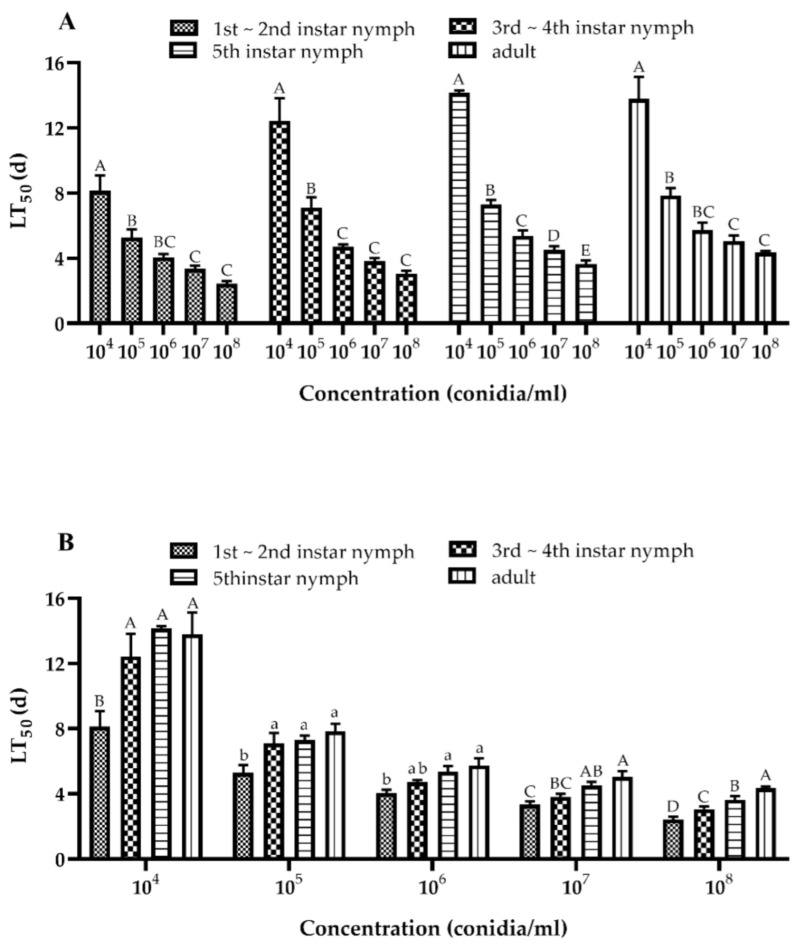
The median lethal time (Means ± SEs) of *D. citri* different developmental stages treated with various concentrations of the *A. fijiensis* GDIZM-1 strain. (**A**). The median lethal time of a conidial suspension with different concentrations applied to *D. citri* at the same developmental stages. (**B**). The median lethal time of conidial suspensions with the same concentration applied to *D. citri* at different developmental stages. One-way ANOVA and Duncan’s new multiple range method were used to analyze the differences among the different treatments. Different lowercase letters indicate that the difference was significant (*p* < 0.05). Different capital letters indicate that the difference was extremely significant (*p* < 0.01).

The results of the lethal time effect of the different conidial concentrations of the *A. fijiensis* GDIZM-1 strain on the nymphs and adults of *D. citri* showed that the LT_50_ of *D. citri* was significantly shortened with increasing concentrations of the *A. fijiensis* GDIZM-1 strain (Figure 7A). The LT_50_ of the nymphs and adults of *D. citri* were different, from long to short: adult > older nymphs (5th instar) > middle nymphs (3rd-4th instar) > younger nymphs (1st-2nd instar) (Figure 7B). These results show that the *A. fijiensis* GDIZM-1 strain had high pathogenicity against *D. citri* and that the mortality of *D. citri* increased with an increasing conidial concentration and treatment time with the *A. fijiensis* GDIZM-1 strain. The LT_50_ and LC_50_ of *D. citri* decreased with an increase in the developmental stage of *D. citri*, during which the *A. fijiensis* GDIZM-1 strain was applied.

#### 3.4.2. Efficacy of *A. fijiensis* and *M. anisopliae* against *D. citri* in Greenhouse Trials

**Figure 8:**
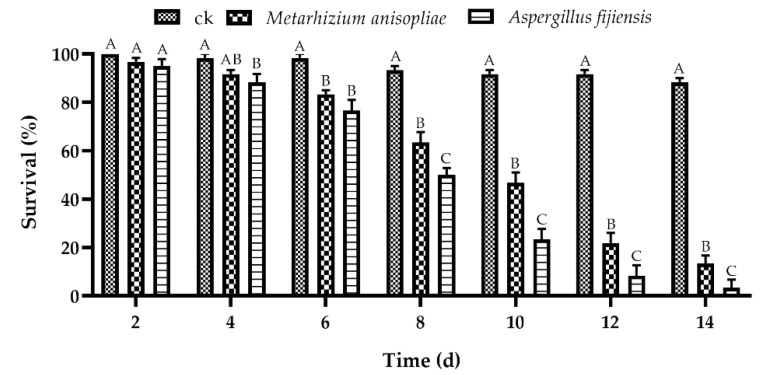
The survival (Means ± SEs) of *Diaphorina citri* adults infected with *Aspergillus fijiensis* GDIZM-1 strain and *Metarhizium anisopliae* GDIZMMa-3 strain (all are 1 × 10^8^ conidia/mL) under greenhouse conditions. One-way ANOVA and Duncan’s new multiple range method were used to analyze the differences among the different treatments. Different capital letters indicate that the difference was extremely significant (*p* < 0.01).

In this study, we evaluated the pathogenicity of the *A. fijiensis* GDIZM-1 strain and the *M. anisopliae* GDIZMMa-3 strain against *D. citri* at 1 × 10^8^ conidia/mL. The results showed that the survival of *D. citri* adults decreased with increasing treatment time (Figure 8). The insecticidal effects of the *A. fijiensis* GDIZM-1 strain and *M. anisopliae* GDIZMMa-3 strain were relatively slow, but gradually increased with time, and the insecticidal effect of the *A. fijiensis* GDIZM-1 strain against *D. citri* was significantly higher than that of the *M. anisopliae* GDIZMMa-3 strain. When infected with the *A. fijiensis* GDIZM-1 strain, the survival of *D. citri* adults was only 3.33% on the 14th day, which was significantly lower than the survival of the *M. anisopliae* GDIZMMa-3 strain and the control (*F* = 259.00, *df* = 2, 6, *p* < 0.001). These results showed that the *A. fijiensis* GDIZM-1 strain had better pathogenicity against *D. citri* adults than the *M. anisopliae* GDIZMMa-3 strain under greenhouse conditions.

## 4. Discussion

*D. citri* is an important vector involved in the natural spread of HLB disease, which is a devastating disease of citrus. Currently, there are no effective control measures for HLB disease, so the prevention and control of *D. citri* and stopping the infection cycle of HLB disease pathogens are important means for the comprehensive management of HLB disease [50,51]. At present, the majority of farmers mainly rely on chemical pesticides for the management of agricultural pests [52], but the side effects of the continuous use of chemical insecticides have attracted people’s attention to biological control. Entomopathogenic fungi are widely found in nature and are a green and safe natural biological resource. Entomopathogenic fungi have become one of the most critical tools to control *D. citri* due to their wide host range and environmental friendliness [53,54].

Most fungi reproduce in sexual and asexual ways, and their sexual spores and surrounding tissue structure are the main basis for the classification of fungi. However, the sexual stage of some fungi degenerates and disappears without forming sporulation structures, and the sexual reproduction stage during growth is not always easy to observe [51]. Therefore, asexual conidial and sporulation structures have become an important basis for the identification of fungal strains [55,56]. However, the morphological characteristics cannot always accurately distinguish closely related species of fungi [57,58]. The ITS gene has been found to be a suitable site for identifying *Aspergillus* genus, but it is not sufficient to distinguish related species so a multi-locus approach is needed [59]. 

In this study, we identified and isolated an entomopathogenic fungus, GDIZM-1, from a cadaver of *D. citri* through field investigation in a lemon orchard. Based on ITS, TEF1 and RPB2 sequences were used to construct a multigene phylogenetic tree, which has a higher reliability than a single-gene phylogenetic tree. Finally, through morphological identification and molecular identification, it was determined that the pathogenic fungus was a new entomopathogenic strain of the species *A. fijiensis*. The colony morphological characteristics and phenotype of the *A. fijiensis* GDIZM-1 strain were very similar to the reported descriptions of *A. fijiensis* strains, and its colony colors, colony textures and conidial surfaces were consistent with the results of Varga et al. [60]. This species was first reported in the USA. It has been found in soil on the Fiji Islands and on *Lactuca sative* in Indonesia and in indoor air, but it has never been reported in China in the past. In addition, it has a substantial economic value, as it includes fermenters of foodstuffs and key cell factories for the production of β-fructofuranosidase [60,61].

The insecticidal mechanisms of entomopathogenic fungi mainly include two types: one is that fungal pathogens proliferate and grow in large numbers after parasitism on healthy insects, absorb nutrients from the host, and finally lead to the death of the host due to lack of nutrition; the other is that the pathogenic fungi produce a variety of toxic metabolites, resulting in the death of the insects by poisoning [9,19]. The toxins and fungal metabolites produced by *Aspergillus* species can also be used to control some pests. For example, mycotoxins, such as aflatoxin B, are toxic to *Periplaneta americana*, and ochratoxin, citrinin and patulin are toxic to *Drosophila melanogaster* [62]. Mensah and Young [63] showed that oil-based extracts of *Aspergillus* species were toxic to *Bemisia tabaci* adults. Kaur et al. [64] reported that an ethyl acetate extract of *Aspergillus niger* adversely affected the survival and development of *Spodoptera litura* and showed antifeedant and toxic effects of *A. niger* metabolites. The results of this study showed that the infected *D. citri* showed slow movement, slight spasms and convulsions and eventually led to death. At present, the cause of death of the infected *D. citri* remains unclear, and further research is needed.

Numerous studies have reported that *Aspergillus* species have high pathogenicity against different insects, indicating that the fungus has promising biological control potential in pest management [32,33,34]. The results of our bioassay showed that with the increase in the conidial concentration of the *A. fijiensis* GDIZM-1 strain, the mortality of *D. citri* increased, and the median lethal time was shortened. It had high pathogenicity against both nymphs and adults of *D. citri*. Its pathogenicity against *D. citri* was comparable to that of *Cordyceps javanica*, *Hirsutella citriformis* and *C. fumosorosea* isolated from *D. citri*, and the growth rate and spore yield are higher than those of *C. javanica* [20,21,65]. This is similar to the control effect of azadirachtin and other commonly used pesticides on *D. citris* [66], so it has the potential to be developed into a new biopesticide resource.

Comparing the virulence results of the *A. fijiensis* GDIZM-1 strain to the nymphs and adults of *D. citri*, it was found that its insecticidal ability against younger nymphs of *D. citri* was higher than that against older nymphs and adults, which may be related to the nutrition and structure of the integument, defense mechanism and microflora composition on the body surface at different developmental stages of *D. citri* [67,68,69]. The nymphs of *D. citri* have weak activity on twigs or buds and are easily infected by fungal spores. Moreover, the honeydew secreted by *D. citri* provides nutrients and favorable conditions for the infection cycle of fungal spores. Therefore, the best opportunity to use entomopathogenic fungi to control *D. citri* is the peak period of younger nymphs to reduce the population of *D. citri* rapidly.

The results of greenhouse trials showed that the pathogenicity of the *A. fijiensis* GDIZM-1 strain against *D. citri* under semifield conditions was lower than that of the indoor effect, which may be related to environmental factors, such as temperature, humidity and ultraviolet radiation [70,71]. Compared with the control effect of the *M. anisopliae* GDIZMMa-3 strain against *D. citri*, the *A. fijiensis* GDIZM-1 strain had significant advantages, which may be due to strain distinctions, host species, temperature, and soil type in various studies [72,73]. Next, it is necessary to verify its specific prevention effect in the field. At the same time, it can also be combined or alternatively used with oil emulsions and chemical pesticides to reduce pesticide residues and pest resistance and achieve the reduction and synergistic effect of pesticides [74].

The green control of pests is the basic concept of pest control in recent years, and the use of entomopathogenic fungi to control agricultural pests has become an important mean. In the present study, the results showed that *A. fijiensis* GDIZM-1 had a good lethal effect against *D. citri* at different developmental stages. Furthermore, researchers found that Aflatoxins belong to a group of toxic and carcinogenic secondary metabolites produced by *Aspergillus* species [75,76,77,78], which pose major health and economic problems worldwide [79,80,81,82]. Aflatoxin B_1_ has been classified as a Group 1 carcinogen by the International Agency for Research on Cancer [83]. Since the aflatoxin B_1_ has strong carcinogenicity and toxicity, these potential risks should be considered before utilizing *A. fijiensis* GDIZM-1 to control *D. citri* near human populations. However, the aflatoxin B_1_ detection results showed that the *A. fijiensis* GDIZM-1 strain is a non-aflatoxin B_1_ producer. The present study provided the first systematic report of an *A. fijiensis* GDIZM-1 strain as an entomopathogenic fungus and a newly discovered pathogen of *D. citri*. We consider that *A. fijiensis* GDIZM-1 has the potential to be developed into a biocontrol agent. Currently, the safety of the *A. fijiensis* GDIZM-1 strain against humans and nontarget organisms is not clear. Therefore, it is necessary not only to evaluate whether it substances harmful to humans and animals and whether it can have a negative impact on the environment and ecology but also to verify its actual control effect in the field. Only after the above issues are clarified can we further develop this new resource and promote the application of biological pesticides.

## 5. Conclusions

In this study, we isolated and identified the *A. fijiensis* GDIZM-1 strain with high pathogenicity against *D. citri* from naturally infected cadavers of *D. citri* adults, which enriches the existing resource library of entomopathogenic fungi. The results of the laboratory and semifield bioassays show that the *A. fijiensis* GDIZM-1 strain has promising biological control effects on *D. citri* and has the potential to be developed into a new biological control agent. Our study is expected to provide a biocontrol option for *D. citri*.

## Figures and Tables

**Table 1:** The reference entomopathogenic fungi used in phylogenetic analysis and their GenBank accession numbers for ITS, TEF1-α and RPB2.

Species	GeneBank Accession Number		
	ITS	TEF1-α	RPB2
*Aspergillus brunneoviolaceus*	MT102843	HE984384	KX650010
*Aspergillus aculeatus*	KY320594	HE984398	MK340898
*Aspergillus japonicus*	KX621981	HE984394	MN969079
*Aspergillus fijiensis*	MH856458	HE984402	HE984375
*Aspergillus fijiensis*	OM925539 *	ON000912 *	ON000911 *
*Aspergillus uvarum*	MZ541955	HE984397	HE984364
*Emericella acristata*	EF652446	KM882998	KU867032

* This is the GenBank accession number of the GDIZM-1 strain.

**Table 2:** Colony diameter and sporulation at Day 10 (means ± SEs) of the *A. fijiensis* GDIZM-1 strain.

Strain	Colony Diameter (mm)	Growth Rate (mm/d)	Sporulation (×10^8^ Conidia/mL)
GDIZM-1	83.70 ± 0.47	16.74 ± 0.10	3.75 ± 0.30

**Table 3:** Regression equations of the pathogenicities of the *Aspergillus fijiensis* GDIZM-1 strain against the different developmental stages of *Diaphorina citri* after 7 days of infection.

Insect Stages	Toxicity Regression Equation	LC_50_ (Conidia/mL)	95% Confidence Interval (Conidia/mL)	χ2	*p*
1st~2nd instar nymph	y=0.30x−1.15	6.40 × 10^3^	5.08 × 10^−1^~7.40 × 10^4^	0.15	0.99
3rd~4th instar nymph	y=0.27x−1.08	1.15 × 10^4^	1.81 × 10^−1^~1.44 × 10^5^	0.22	0.98
5th instar nymph	y=0.28x−1.43	1.20 × 10^5^	0.86 × 10^3^~9.76 × 10^5^	0.08	0.99
Adult	y=0.28x−1.52	2.77 × 10^5^	5.45 × 10^3^~2.48 × 10^6^	0.48	0.92

## Data Availability

The raw data supporting the conclusions of this manuscript will be made available by the authors, without undue reservation, to any qualified researcher upon request.
